# Ozone oxidative preconditioning inhibits renal fibrosis induced by ischemia and reperfusion injury in rats

**DOI:** 10.3892/etm.2014.2004

**Published:** 2014-10-06

**Authors:** LEI WANG, HUI CHEN, XIU-HENG LIU, ZHI-YUAN CHEN, XIAO-DONG WENG, TAO QIU, LIN LIU, HENG-CHENG ZHU

**Affiliations:** Department of Urology, Renmin Hospital of Wuhan University, Wuhan, Hubei 430060, P.R. China

**Keywords:** ozone oxidative preconditioning, renal fibrosis, ischemia and reperfusion injury

## Abstract

Ischemia and reperfusion injury (IRI) is a crucial contributor to the development of renal fibrosis. Ozone has been proposed as a novel medical therapy for various conditions, including organ IRI. The aim of this study was to investigate whether ozone oxidative preconditioning (OzoneOP) has a beneficial effect in preventing the development of renal fibrosis following IRI. Sprague Dawley rats were subjected to 45 min of ischemia followed by 8 weeks of reperfusion. Prior to surgery, rats in the OzoneOP group were treated with ozone and those in the IRI and Sham groups were untreated. Blood samples were collected for the detection of blood urea nitrogen (BUN) and creatinine (Cr) levels. To assess tissue fibrosis, Masson’s trichrome staining was performed. Immunohistochemistry was also performed to determine the localization of α-smooth muscle actin (α-SMA). Reverse transcription quantitative polymerase chain reaction (RT-qPCR) and western blotting were conducted to analyze the expression of transforming growth factor (TGF)-β1, α-SMA and Smad7. The levels of BUN and Cr did not significantly differ between groups. Rats pretreated with ozone showed markedly less interstitial fibrosis than untreated rats following IRI. In addition, immunohistochemistry revealed that α-SMA expression was attenuated in the OzoneOP group compared with the IRI group. RT-qPCR and western blot analysis showed that OzoneOP inhibited the IRI-induced increases in α-SMA and TGF-β1 expression levels, and that the IRI-induced reduction in the expression of Smad7 was inhibited in the OzoneOP group. The results indicate that OzoneOP has beneficial effects on ischemic renal fibrosis. OzoneOP may exert its protective effects by a mechanism involving modulation of the TGF-β1/Smad7 pathway.

## Introduction

Renal ischemia and reperfusion injury (IRI) is a common cause of renal failure and often occurs in surgeries such as kidney transplantation, renal artery angioplasty, partial nephrectomy, accidental or iatrogenic trauma, hydronephrosis and elective urological surgery (1,2). In kidney transplantation, renal IRI can lead to acute kidney injury (AKI), which is a syndrome with an abrupt loss of renal function (3). It is also a significant contributor to graft tubular atrophy and interstitial fibrosis, the main cause of graft loss occurring more than one year after transplantation (4).

Renal fibrosis is the major pathological change that drives kidney diseases to the end stage (5), and is characterized by glomerulosclerosis, tubulointerstitial fibrosis and tubular atrophy and dilation. In previous studies, transforming growth factor-β1 (TGF-β1) has been identified to play a key role in renal tubular interstitial fibrosis (6,7). TGF-β1 may initiate the transition from renal tubular epithelial cells to myofibroblasts, which is also the cellular source for extracellular matrix (ECM) deposition (8). Smad7, an intracellular signaling mediator of TGF-β family members, plays a pivotal role in TGF-β1 signal transduction (9). A large number of *in vitro* and *in vivo* studies have found that Smad7 antagonizes TGF-β1 by degrading activated receptor complexes (10,11).

Ozone oxidative preconditioning (OzoneOP) is a novel treatment to protect organs from IRI and is relatively simple and harmless. A few studies (12,13), including one from authors of the present study (14), have focused on the protective phenomenon of OzoneOP against inflammation, apoptosis and oxidative stress in rat models of IRI. However, to the best of our knowledge, there have been no reports concerning the role of OzoneOP in renal fibrosis following IRI. In the present study, the role of OzoneOP in ischemia-induced renal fibrosis was investigated. Moreover, the IRI-associated molecules α-smooth muscle actin (α-SMA), transforming growth factor (TGF)-β1 and Smad7 were tested to determine whether and how OzoneOP affected the renal fibrosis.

## Materials and methods

### Animal preparation

All adult male Sprague Dawley rats (220–250 g) were from the Center of Experimental Animals in the Medical College of Wuhan University (Wuhan, China). This project was approved by the Committee on the Ethics of Animal Experiments of Wuhan University, and the procedures were carried out according to routine animal care standards. All experimental procedures complied with the Guidelines for the Care and Use of Laboratory Animals (National Academy Press, 1996). Briefly, rats were anesthetized with pentobarbital (45 mg/kg) and placed on a homeothermic table in order to maintain the core body temperature at 37°C. A midline laparotomy was made and right nephrectomy was performed. Subsequently, the left kidney was subjected to 45 min of ischemia followed by reperfusion.

The animals were divided into sham-operated (Sham), IRI and OzoneOP groups. Each group contained 8 rats. In the Sham group, the surgery involved only removal of the right kidney. In the IRI and OzoneOP groups, the left kidney vessels were clamped for 45 min followed by reperfusion. In the OzoneOP group, prior to surgery, the rats received 15 OzoneOP treatments by rectal insufflation (1 mg/kg), once a day, as previously described (11). The ozone concentration was 50 μg/ml. At 8 weeks after IRI, the left kidneys were removed for analysis and blood samples were collected for the detection of blood urea nitrogen (BUN) and creatinine (Cr) levels.

### Preservation of kidneys

The left kidney was removed under fully maintained anesthesia. After removal, the kidney was fixed in 10% phosphate-buffered formalin or immediately frozen, and stored at −80°C for following experiments.

### Serum assays

At 8 weeks after the surgery in each group, 1-ml blood samples were taken and assays were performed according to the instructions of commercially available creatinine and urea assay kits (Nanjing Jiancheng Bioengineering Research Institute, Nanjing, China). The absorbance was measured by spectrophotometry using a Shimadzu UV-1700 spectrophotometer (Kyoto, Japan; absorbance measured at 510 and 640 nm for the creatinine and urea assay kits, respectively) and the concentrations of BUN and Cr were calculated.

### Masson’s trichrome staining

Following fixation of the kidney in 10% phosphate-buffered formalin, it was embedded with paraffin and cut into 5-μm sections. The sections were deparaffinized and hydrated gradually, and stained with Masson’s trichrome. Morphologic assessments were observed by an experienced renal pathologist who was unaware of the groups and treatments.

### Immunohistochemistry

The expression of α-SMA was conducted by immunohistochemical staining. Briefly, 5-μm sections were deparaffinized, and endogenous peroxidase activity was blocked with 3% hydrogen peroxide at 37°C for 10 min. Then, the sections were treated with 10% normal goat serum (Boster Biological Technology, Ltd., Wuhan, China) in Tris-buffered saline (TBS) for 30 min at 37°C. Subsequently, they were incubated overnight at 4°C with a rabbit polyclonal to α smooth muscle actin antibody (α-SMA; dilution at 1:100 for immunohistochemistry; ab5694; Abcam, Cambridge, MA, USA). After washing three times with phosphate-buffered saline (PBS), these sections were incubated with the secondary antibody (ZSGB-BIO Co., Beijing, China) for 30 min at room temperature, followed by color reagent 3,3′-diaminobenzidine (DAB). For the negative control group, the procedures were performed with the exception of the addition of the primary antibody.

### Reverse transcription quantitative polymerase chain reaction (RT-qPCR)

Total RNA was isolated using TRIzol reagent (Invitrogen Life Technologies, Carlsbad, CA, USA) and the RNA concentration was determined spectrophotometrically. Single-stranded cDNA was synthesized using the cDNA synthesis kit (Takara Bio Inc., Kyoto, Japan) according to the manufacturer’s instructions. RT-qPCR was performed with the Platinum^®^ SYBR^®^ Green qPCR SuperMix-UDG kit (Applied Biosystems, Foster City, CA, USA). The primers used were as follows: α-SMA forward, 5′-CAACCCCTATACAACCATCACAC-3′, and α-SMA reverse, 5′-CCCAAACTGCTTGCGTAACC-3′ (GenBank accession number NM_031005); TGF-β1 forward, 5′-CTTTAGGAAGGACCTGGGTTG-3′, and TGF-β1 reverse 5′-GGTTGTGTTGGTTGTAGAGGG-3′ (GenBank accession number NM_021578); Smad7 forward, 5′-GGCTTTCAGATTCCCAACTTC-3′, and Smad7 reverse, 5′-CGCCATCCACTTCCCTTGT-3′ (GenBank accession number NM_030858). β-actin was used as a housekeeping gene. The data were presented as a ratio of the gene to β-actin mRNA (sense: 5′-TGCTATGTTGCCCTAGACTTCG-3′ and antisense: 5′-GTTGGCATAGAGGTCTTTACGG-3′; GenBank accession number NM_031144). The initial activation was at 95°C for 15 sec followed by 58°C for 20 sec and 72°C for 20 sec, cycling 40 times. The SLAN-96S Real-Time PCR system (Shanghai Hongshi Medical Technology Co., Ltd, Shanghai, China) was used. Three samples were used per assay.

### Western blot

Total proteins were extracted, using RIPA buffer and a protease inhibitor, and quantified using the bicinchoninic acid method. Then, equivalent weights of protein (40 μg/lane) were separated on 10% SDS-PAGE gels and transferred to a nitrocellulose membrane. The membranes were blocked with 5% non-fat milk in Tris-buffered saline and Tween 20 (TBST) buffer and then incubated with the following rabbit anti-rat polyclonal primary antibodies: α-SMA (1:1,000 dilution; Abcam; ab5694), TGF-β1 (1:1,000 dilution; Santa Cruz Biotechnology, Inc.; sc146) and Smad7 (1:1,000 dilution; Santa Cruz Biotechnology, Inc.; sc11392). Subsequently, after washing twice with PBS, the membranes were incubated with secondary antibody (ZSGB-BIO Co.) conjugated with horseradish peroxidase at 1:2,000 dilution. Specific bands were visualized using an Immobilon Western Chemiluminescent HRP Substrate kit (Millipore, Darmstadt, Germany).

### Statistical analysis

Data were presented as the mean ± standard error of the mean. The means of the different groups were compared using one-way analysis of variance (ANOVA) and Student-Newman-Keuls tests. Differences were considered statistically significant when P<0.05.

## Results

### Long-term renal function outcomes

**Figure 1:**
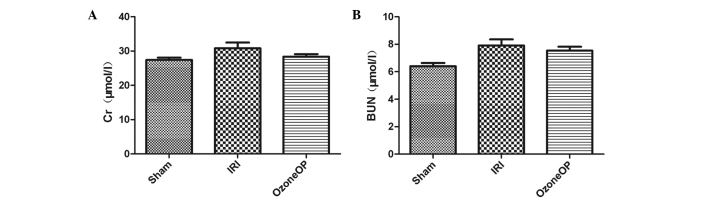
Effects of OzoneOP modification on renal function. Effects of OzoneOP on (A) serum Cr concentrations and (B) serum BUN concentrations after 45 min of ischemia followed by 8 weeks of reperfusion. Bars represent the mean ± standard error of the mean (n=8). OzoneOP, ozone oxidative preconditioning; Cr, creatinine; BUN, blood urea nitrogen; IRI, ischemia and reperfusion injury.

BUN and Cr levels were measured at 8 weeks following IRI in rats that were treated with OzoneOP or were untreated. In the IRI model, renal function was not altered significantly from that in the Sham group and the preconditioning treatment with ozone did not change these results (Fig. 1).

### Morphologic features

**Figure 2:**
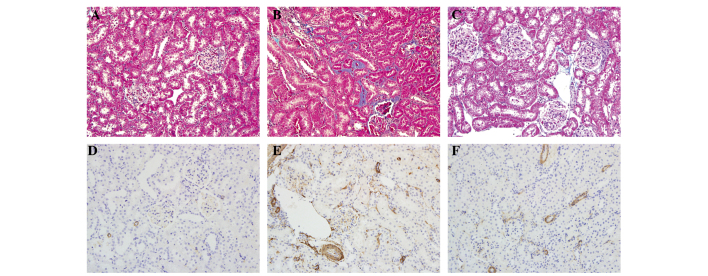
Morphologic features revealed using Masson’s trichrome staining (A–C) and the expression of α-SMA in the kidneys revealed by immunohistochemistry (D–F). Sections from (A and D) a sham-operated rat, (B and E) a rat subjected to IRI, and (C and F) a rat subjected to OzoneOP. Original magnification ×200. SMA, smooth muscle actin; IRI, ischemia and reperfusion injury; OzoneOP, ozone oxidative preconditioning.

Morphologic features were evaluated using Masson’s trichrome staining (Fig. 2A–C). In the Sham group, it revealed little deposition of collagen in the renal cortical tissue sections; however, a significant increase in tubulointerstitial collagen deposition was observed in rats subjected to IRI. In the OzoneOP group, an increase in collagen staining compared with that in the Sham group was observed; however, the staining was less than that observed in the IRI group.

### Localization of the expression of α-SMA

The localization of α-SMA was observed by immunohistochemistry. Staining revealed that α-SMA was rarely found in the Sham group. However, in the IRI group, renal tissues were strongly positive for α-SMA expression, which was mainly localized in the injured renal tubular epithelial cells, tubulointerstitium and vascular smooth muscle. The α-SMA expression in the OzoneOP group was reduced compared with that in the IRI group (Fig. 2D–F).

### RT-qPCR analysis

**Figure 3:**
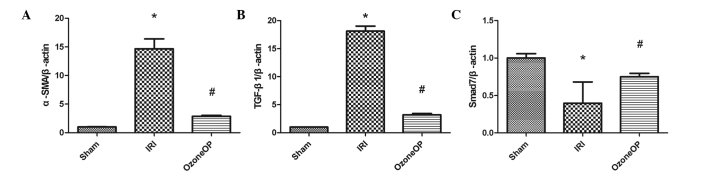
mRNA levels of α-SMA, TGF-β1 and Smad7 in the rat kidney. Effects of OzoneOP on (A) the mRNA level of α-SMA, (B) the mRNA level of TGF-β1 and (C) the mRNA level of Smad7 after 45 min of ischemia followed by 8 weeks of reperfusion. mRNA was standardized for β-actin mRNA. Bars represent the mean ± standard error of the mean. ^*^P<0.05 versus the Sham group; ^#^P<0.05 versus the IRI group. SMA, smooth muscle actin; TGF, transforming growth factor; OzoneOP, ozone oxidative preconditioning; IRI, ischemia and reperfusion injury.

To investigate the mRNA levels of α-SMA, TGF-β1 and Smad7, RT-qPCR analyses were conducted. The expression levels of α-SMA, TGF-β1 and Smad7 relative to β-actin were determined. The mRNA levels of α-SMA and TGF-β1 were significantly greater in the IRI group than in the Sham group. However, OzoneOP treatment inhibited their expression following renal IRI. The mRNA level of Smad7 was reduced in rats subjected to IRI compared with that in the Sham group, and OzoneOP attenuated the reduction (Fig. 3).

### Western blot analysis

**Figure 4:**
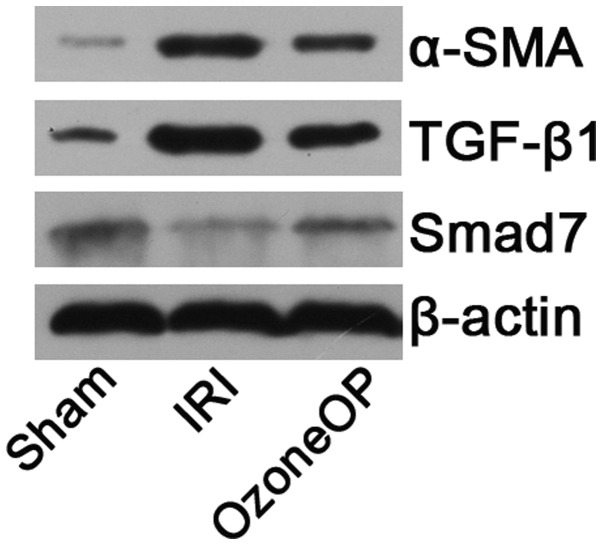
Representative Western blots showed the effects of OzoneOP on α-SMA, TGF-β1 and Smad7 expression in the kidney after 45 min of ischemia followed by 8 weeks of reperfusion. β-actin was used to show equal amounts of protein loading in each lane. SMA, smooth muscle actin; TGF, transforming growth factor; OzoneOP, ozone oxidative preconditioning; IRI, ischemia and reperfusion injury.

The results of western blot corroborated the RT-qPCR findings. The expression levels of α-SMA and TGF-β1 were upregulated in the IRI and OzoneOP groups when compared with those in the Sham group. However, OzoneOP attenuate the expression induced by IRI. Smad7 expression was downregulated in rats subjected to IRI compared with that in the Sham group. However, in the OzoneOP group, the expression level of Smad7 was clearly greater than that observed in the IRI group (Fig. 4).

## Discussion

Ozone has been investigated as a novel treatment for a number of diseases (15,16). In one study, it was demonstrated that ozone had protective anti-apoptosis and anti-inflammatory effects in an animal model of organ IRI (14). Ozone may be used therapeutically to protect organs from IRI and is relatively simple and harmless. It has also been shown to improve the functioning of organs subjected to IRI by inducing them to adapt to slight and transient oxidative stresses, resulting in improvements of endogenous antioxidant systems (17).

IRI is of significant interest to researchers due to its impact on such organs as the kidney, liver and heart. It is the leading antigen-independent factor contributing to the development of chronic allograft loss, which is the foremost cause of graft loss occurring more than one year after kidney transplantation (18). In addition, it is also a crucial contributor to renal fibrosis, which is characterized by glomerulosclerosis, rarefaction of the glomerular and peritubular capillaries, and tubulointerstitial fibrosis (19). The long-term inflammation elicited by IRI can result in fibrosis. Therefore, it is essential to find alternative strategies to counteract the development of fibrotic tissue. In a previous study, we demonstrated that OzoneOP has renal protective effects associated with its anti-apoptosis and anti-inflammatory properties (14). In the present study, the aim was to investigate whether OzoneOP could have a beneficial effect in preventing the development of renal fibrosis following IRI and to elucidate the underlying mechanism.

Prior to 45 min of ischemia, certain rats received 15 ozone preconditioning treatments by rectal insufflation, and subsequently, they underwent reperfusion for up to 8 weeks. Although renal function showed no significant differences among all groups, the results of Masson’s trichrome staining showed that IRI induced a significant increase in tubulointerstitial collagen deposition in rats subjected to IRI. However, this pathologic finding of renal fibrosis was significantly ameliorated by OzoneOP. In addition, previous studies have confirmed that OzoneOP is able to reduce the short-term inflammatory response following organ IRI (12,14). In the current study, observation of Masson’s trichrome staining revealed extensive inflammatory cell infiltration in the IRI group. However, inflammatory cells were sparsely distributed in the OzoneOP group. These results indicate that OzoneOP suppresses long-term inflammation and thereby attenuates the development of renal fibrosis.

TGF-β1 has been demonstrated to be not only a multipurpose cytokine but also a crucial inducer of renal fibrosis (20). It increases ECM deposition by enhancing the synthesis of ECM proteins and inducing the protease inhibitors blocking their degradation (21). Using animal models, increased expression of TGF-β1 has been found to be universal in various kinds of chronic kidney disease. The results of *in vitro* studies are also in accordance with those of *in vivo studies*. TGF-β1 may induce interstitial fibroblasts and tubular epithelial cells to undergo epithelial-to-mesenchymal transition (EMT) to become matrix-producing fibrogenic cells. The expression of α-SMA parallels that of TGF-β1. α-SMA is often expressed during EMT and it has been viewed as a marker of ‘activated’ fibroblasts. As mediators of TGF-β1 family members, Smad proteins are important molecules in its signal transduction pathway (22,23). In the present study, it was found that the expression of TGF-β1 and α-SMA was upregulated in the IRI and OzoneOP groups due to IRI; however, the changes induced by IRI were attenuated significantly in the OzoneOP group. Furthermore, the expression of Smad7 in the IRI group showed a significant reduction when compared with that in the Sham group, and OzoneOP markedly inhibited this reduction. This indicates that OzoneOP affects the expression of TGF-β1/Smad7 and thereby exerts protective effects against the renal fibrosis induced by IRI.

To the best of our knowledge, the present study is the first to demonstrate that OzoneOP is able to protect the ischemic kidney against renal fibrosis. This protective effect may be achieved via modulation of the TGF-β1/Smad7 pathway. Therefore, these findings reveal the potential role of ozone as a novel therapeutic option against ischemic renal fibrosis.
